# Refining a primary care shared decision-making aid for lifestyle change: a mixed-methods study

**DOI:** 10.3399/BJGPO.2021.0100

**Published:** 2022-02-23

**Authors:** Neil Heron, Seán R O’Connor, Frank Kee, David R Thompson, Margaret Cupples, Michael Donnelly

**Affiliations:** 1 Centre for Public Health, Queen’s University Belfast, Belfast, Northern Ireland; 2 School of Primary, Community and Social Care, Keele University, Keele, UK; 3 School of Psychology, Queen’s University Belfast, Belfast, Northern Ireland; 4 School of Nursing and Midwifery, Queen’s University Belfast, Belfast, Northern Ireland

**Keywords:** decision making, shared, lifestyle, primary care, general practice

## Abstract

**Background:**

The important role of primary care in promoting healthy lifestyle behaviours needs informed support.

**Aim:**

To elicit views on a 39-item shared decision-making (SDM) aid (SHARE-D) for lifestyle change and refine it to improve implementation.

**Design & setting:**

Mixed-methods study.

**Method:**

Health professionals, patients, and support workers, with experience of managing or a history of cardio- or cerebrovascular disease, were purposively recruited based on age, sex, and urban/rural location (*n* = 34). Participants completed a survey, rating the importance of including each item in a decision-aid, designed for use by patients with health professionals, and suggesting modifications. Semi-structured interviews (*n* = 30/34) were conducted and analysed thematically.

**Results:**

Substantial agreement was observed on rating item inclusion. Based on survey and interview data, 9/39 items were removed; 13 were amended. Qualitative themes were: 1) core content of the decision-aid; 2) barriers to use; 3) motivation for lifestyle change; and 4) primary care implementation. ‘Self-reflective’ questions and goal setting were viewed as essential components. The paper-based format, length, clarity, and time required were barriers to its use. Optional support considered within the aid was seen as important to motivate change. A digital version, integrated into patient record systems was regarded as critical to implementation. A revised 30-item aid was considered suitable for facilitating brief conversations and promoting patient autonomy.

**Conclusion:**

The SHARE-D decision aid for healthy lifestyle change appears to have good content validity and acceptability. Survey and interview data provided in-depth information to support implementation of a refined version. Further studies should examine its effectiveness.

## How this fits in

SDM can ensure that patients’ individual care needs and preferences are met. There is limited evidence about using these approaches for promoting behaviour change, or how they could work best in general practice. This mixed methods study explored the views of patients and health professionals on an existing decision-making aid. Findings highlighted how the aid could be used to structure brief conversations about lifestyle change. Recommendations are made to support its use in primary care.

## Introduction

Routine consultations in primary care settings present an important opportunity to target lifestyle behaviours.^1^ Current UK guidelines recommend that health professionals identify patients who are not sufﬁciently active and offer support to encourage behaviour change.^2^ Lifestyle advice can be of particular benefit to patients with established cardiovascular disease risk factors, including physical inactivity, poor diet, and obesity.^3–6^ However, these factors are infrequently discussed with patients in routine practice.^7^ This is often attributed to limited organisational resources and time, as well as a need to address other issues during consultations.^8,9^ Effective, novel methods of delivery may be required to address these constraints.^10^

Previous studies on the effectiveness of offering lifestyle advice in primary care have shown that though increases in physical activity can occur, these are short-term, and not sufficient to enable sustained behaviour change.^11^ In a qualitative study of a tailored intervention to promote secondary cardiovascular risk reduction in general practice, the authors found many participants made only brief attempts at lifestyle changes.^12^ Discussions on lifestyle change can be challenging to initiate, particularly around issues such as weight reduction,^13^ and practitioner–patient priorities and expectations of conversations can also differ.^9,14^ SDM is a process in which clinicians actively involve patients to ensure support meets their individual preferences.^15–17^ Decision aids have focused primarily on treatment or health screening options.^18–20^ Less is known regarding their use in promoting behaviour change. Further, there is limited evidence that has reported on user-centred, iterative development of SDM aids,^21^ which is imperative for decision-aids used for managing chronic conditions. An additional recommendation for the systematic development of decision aids is that they include a strong theoretical underpinning,^22^ drawing on approaches including the 5A’s model of brief behavioural counselling^23,24^ and the COM-B framework,^25,26^ which accounts for the influence of context on behaviour through consideration of capabilities, opportunities, motivation, and social, physical, and environmental factors.^26^ The COM-B framework informed the design and development of SHARE-D,^27^ a questionnaire-based aid, designed to facilitate SDM around lifestyle modification in patients with increased cardiovascular risk factors and to identify constraints and support that may be needed for making changes to diet and physical activity.

A feasibility study using SHARE-D in primary care identified that both patients and practitioners valued its use, felt it helped structure consultations, and provided the opportunity to discuss benefits of and difficulties associated with making lifestyle changes.^27^ However, due to the time taken to use the aid, a need for further user-centred development and modification was identified before it could be effectively used in everyday practice. The aim of this study was to explore views on SHARE-D and gain consensus on how it should be refined to improve its implementation in primary care settings.

## Method

A convergent mixed-methods approach^28^ was used to combine online survey data with findings from semi-structured interviews. Recommendations of the consolidated criteria for reporting qualitative research (COREQ) were followed for the interview component of the study.^29^

### Participants

GPs, practice nurses, doctors working in cardiovascular medicine, healthcare professionals working in cardiac rehabilitation settings, support workers, and patients from cardiac and stroke support groups were eligible to participate in the study. The study team were provided with lists of eligible individuals from primary care settings within five NHS Trusts in Northern Ireland, academic primary care departments, two cardiovascular clinical interest groups, and a patient support group linked to a national charity (Northern Ireland Chest Heart and Stroke). Purposeful sampling was used to ensure a balance between key participant characteristics (age, sex, professional group, or patient history) and 75 people were invited to take part via e-mail or telephone call. In line with recommendations around optimal sample sizes, the study aimed to recruit 30 individuals based on the expected heterogeneity of responders from the patient and health professional groups.^30^ No exclusions were applied to age, duration of treatment (for patient participants), or years of clinical experience (for GPs or other health professionals). Informed consent was obtained prior to participation.

### Data collection

Participants were sent an electronic copy of the SHARE-D aid and a description of its purpose (see Supplementary Figure S1). They were asked to review the aid and then complete an online survey stating if they agreed that each item ‘should be included in the aid’, and ‘was clear and understandable’ (both responses were assessed as ‘agree’, ‘disagree’, or ‘unsure’ for all 39 items). Participants were asked to suggest any improvements or modifications to each item. Within 4 weeks of the survey, participants were contacted to arrange a semi-structured interview. These were conducted by an experienced researcher and clinician with a background in health services research. All interviews were carried out remotely by telephone or video-conferencing facilities during May and June 2020. An interview schedule, consisting of open-ended questions, was used (see Table 1). This was based on previous research,^27^ and was reviewed and adapted prior to the interviews based on survey findings. Participants’ individual survey responses and the aggregated responses of the sample were discussed. Interviews also explored views on proposed changes to items, as well as perspectives on discussing lifestyle change in primary care. Interviews were audiorecorded and transcribed verbatim. Field notes were recorded. These were summarised to support the analysis and interpretation of data. All participants were informed that they could request a copy of the final analysis, if they wished to review it.

**Table 1.** Interview schedule

1. Can you tell me about any experience you have of discussing lifestyle changes with a healthcare professional, a friend or family member? (Patient)
2. Can you tell me about any experience you have of using a ‘discussion tool’ or a ‘shared decision aid’?
3. What would your expectations of such a tool be?
4. Are these expectations met by the SHARE-D aid?
5. What parts of the SHARE-D tool did you like?
6. What would you want to change about the SHARE-D aid?
7. Did you learn anything new from the SHARE-D aid? Were there any surprises?
8. Is there anything else you would like the SHARE-D aid to include?
9. How do you think using the aid might affect lifestyle behaviours like physical activity and diet?
10. What do you think about the activities and questions included in the SHARE-D aid?
11. What would help people to remember what was discussed after using the SHARE-D aid?
12. How confident would you be that people using the aid could stick to any lifestyle decisions made when using the SHARE-D aid?
13. Who do you think is best placed to use the SHARE-D aid?
14. What do you think is the best setting for the SHARE-D aid to be used?
15. What do you think is the best format for the SHARE-D aid?
16. What would the benefits and disadvantages be of an online version of the SHARE-D aid?
17. What do you think about how long the conversation guided by the SHARE-D aid should be?
18. Is there anything else you would like to say about the SHARE-D aid?

### Data analysis

Online survey data were extracted into Microsoft Excel and percentage agreement scores were calculated in respect of understandability of each item and if it should be included. Items with <80% agreement on either understandability or inclusion were identified and selected for discussion during interviews to determine if they should be removed from a revised version of the SHARE-D aid. Consensus was considered to have been met at 80%, based on previous recommendations for establishing agreement between expert panel members.^31^ Individual item and overall agreement between raters on inclusion of items was determined using free-marginal multirater Kappa scores for categorical variables (Randolph’s K).^32^ For the interview data, a reflexive thematic analysis was used based on an inductive approach.^28^ Transcribed data underwent a five-stage process during analysis. This included data familiarisation, coding, generation of initial themes, review and definition of themes, and an analytical narrative synthesis to contextualise findings. Data were coded using NVivo (version 12.0). Two authors cross-coded transcripts and met to discuss and resolve any disagreement. All decisions were discussed and confirmed via consensus between authors.

## Results

Characteristics of the 34 participants (12 GPs, seven other health professionals, 11 patients, and four support workers) are shown in Table 2. The online survey took an average of 43.5 minutes to complete (range 29–61 minutes; standard deviation 10.3). Overall percentage agreement on rating whether all 39 items in the aid should be included was 74.9%, corresponding to a substantial level of agreement (K = 0.62; 95% confidence interval = 0.54 to 0.71). A substantial level of agreement was seen between participants from a health professional background, with moderate agreement among patients and support workers (see Table 3). Agreement for inclusion of individual items ranged from 20.6% for one of the images in the aid (K = 0.06; slight agreement) to 100% for the item asking ‘is being active important to you’ (K = 1.00; almost perfect agreement [see Supplementary Table S1]). Based on individual percentage agreement scores, 11/39 items (28%) were rated as candidates for removal from the aid (<80% agreement on inclusion); these included pictorial images and items viewed as providing no additional value.

**Table 2.** Participant characteristics

**Characteristic**	**Health professionals (*n* = 19**), *n*	**Patients and support workers (*n* = 15**), *n* ^a^	**Total sample (*n* = 34**), *n*
Age, years			
18–34	4	0	4
35–54	8	3	11
55–64	5	7	12
≥65	2	5	7
Sex			
Male	7	6	13
Female	12	9	21
Location			
Urban	8	9	17
Semi-rural/rural	11	6	17
Profession			
GP	12	—	12
Cardiac physiotherapist	5	—	5
Practice nurse	1	—	1
Cardiovascular medicine physician	1	—	1
Condition^b^			
Heart disease	—	9	9
Stroke/TIA	—	3	3

^a^Four support workers were included; one of whom also had a previous cardiovascular event. ^b^Heart disease or TIA/stroke. TIA = transient ischaemic attack.

**Table 3.** Overall level of agreement on inclusion of each item (*n* = 39) in the SHARE-D aid

Category	**Overall agreement on inclusion of each item, %**	**Overall agreement on inclusion (Randolph’s K**)	**95%** CI	**Level of agreement^a^**	***P* value**
Overall agreement for sample (*n* = 34)	74.9	0.62	0.54 to 0.71	Substantial	0.001
Health professional agreement (*n* = 19)	76.4	0.68	0.60 to 0.76	Substantial	0.001
Patient and support worker agreement (*n* = 15)	73.4	0.58	0.47 to 0.65	Moderate	0.001

Level of agreement based on the following categories: <0: poor agreement; 0.0–0.20: slight agreement; 0.21–0.40: fair agreement; 0.41–0.60: moderate agreement; 0.61–0.80: substantial agreement; 0.81–1.0: almost perfect agreement.

Four participants (two patients and two health professionals) could not be contacted within the 4-week time frame, meaning that 30 interviews were completed (88.2% of the sample). Interviews lasted approximately 35–45 minutes. Four overarching themes emerged from the thematic analysis of the qualitative data. These were: 1) core content of the decision aid; 2) barriers to use; 3) motivating factors for lifestyle change; and 4) implementation in primary care. These themes are described below. Illustrative quotes are included in Table 4 to provide context.

**Table 4.** Selected participant quotes related to each theme identified based on analysis of qualitative interview data (*n* = 30 interviews)

Views of participants	Related theme	Supporting quote(s)
Should ensure conversations were ‘routine and non-personalised’	Theme 1. Core content of the decision aid	‘*I like that you can speak about the need to make changes, but can back this up with the advice from guidelines, you know, it’s not just me saying this. Patients understand who NICE* [National Institute for Health and Care Excellence] *are and are willing to listen to this.’* (Health professional 9, female)
Participants in favour of ensuring that there was a ‘quick pathway’ through the aid; continuity of care could be difficult; patients’ readiness to make lifestyle changes may vary over time; and identifying those willing to engage could be difficult	Theme 2. Barriers to use	‘… *there could be a role for a longer version used where time allowed, or if used outside of a typical 10-minute primary care consultation.’* (GP 7, female) ‘*I would think going back would be good, you could use it* [the appointment] *to check what you are or have been doing, but I guess you might not see the same doctor and you would just be starting again with someone else.’* (Patient 3, male) ‘*Going through the tool without any engagement from the patient could be time consuming, unnecessary, and even detrimental to other aspects of care’* (Health professional 3, female) ‘... *even in two or three minutes you might sometimes have an impact* [on patients] *but usually the ability to spend time to do this is restricted, or is restricted if patients really are only there for you to send them off with their tablets.’* (GP 4, male)
Information could help prepare people for change, but more support might be needed to motivate them	Theme 3. Motivating factors for lifestyle change	‘... *I know what the benefits are, but it’s the next steps that are important, I think actually sticking to changes is much harder, and there is always something getting in the way.‘* (Patient 5, female)
Even brief information could be useful to patients at a later time	Theme 4. Implementation in primary care	‘… *people might not be ready to make changes at that moment, but they need to be reassured about the safety of slowly starting to exercise.’* (Patient support worker 2, male)
Patients could go through the aid themselves, prior to a consultation	—	‘*I think sending a link to the tool* [before an appointment] *could be very useful, when you have spoken to the patient about the issues, you could use that opportunity to follow up on that quite quickly. It makes me think about text-messaging systems we use for patients.’* (GP 3, female)

### Theme 1. Core content of the decision aid

SHARE-D was perceived to have the potential to improve patient outcomes by providing a guide to facilitating brief, focused discussions about a healthy lifestyle, communicating guideline-informed advice clearly, and supporting behaviour change. Referring to improved outcomes if physical activity and dietary guidelines are followed, and highlighting guidelines, was perceived as important to ensure conversations were ‘routine and non-personalised’.

Although participants valued the questions included in the aid, most underlined that there were too many. It was highlighted that some were useful in helping patients to ‘reflect on’ decisions about lifestyle change. This was seen as important by GPs who suggested that a role of the aid should be to promote greater patient autonomy. The most commonly endorsed component that was important to retain in the aid was goal setting. Participants’ views indicated a need to add examples of goal setting that they could consider after a conversation with their GP, and that they could use to set further goals themselves, with the aim of motivating them to sustain these lifestyle changes.

### Theme 2. Barriers to use

Health professionals stated that while the paper-based format of the aid was useful, it could be impractical. Some cited limited space for storage of paper booklets or underlined the fact that working in different offices meant that they had to carry materials and equipment with them. The main barrier identified was the lack of time during consultations to use the aid in its current format. Participants reported that the number of items could be reduced to facilitate brief conversations, and that this could be done without compromising the key messages. Participants were in favour of ensuring that there was a ‘quick pathway’ through the aid, but acknowledged that a longer version could be feasible if time was not constrained.

The support options provided were seen as important (providing written information and discussing options with others) but a frequently expressed patient view was that reviewing discussions in a follow-up consultation would be beneficial. It was also acknowledged that continuity of care could be difficult as patients would not always be able to see the same GP. Current practice in promoting healthy lifestyle behaviour involves providing patients with evidence-based information sheets (for example, from web-based resources such as https://patient.info), which could then be recorded in patient records. Some participants considered that the lack of integration of the SHARE-D aid and a record of its use in current electronic record systems was a barrier to its use.

Consultations arranged with an intended focus on current physical or psychological problems, rather than on lifestyle behaviours, were also identified as barriers to engagement with the aid. In addition, participants reported there was a limit to how much information could be provided and taken in during a single appointment, and that a system for providing summarised information or reminders would be a useful addition. Patients highlighted how lack of clarity around terms, including ‘decision aid’, could be an issue. Although physical activity guidelines were seen as an important component, participants were aware that meeting these recommendations could be challenging, and that missing targets, or not seeing benefit from changes to lifestyle, could be demotivating. Participants recognised that patients’ readiness to make lifestyle changes may vary over time and suggested that identifying those willing to engage in healthy lifestyle discussion and changes could be difficult.

### Theme 3. Motivating factors for lifestyle change

Patients reported that information on benefits of lifestyle change could help prepare people for change, but highlighted that more support might be needed to motivate them to make changes. Another issue was how some questions could be better framed to avoid negative responses, which could otherwise fail to motivate engagement with the aid, as well as with lifestyle change. For example, the statement, ’I need other people to help me make lifestyle changes’, could be rephrased as ’asking others for support can help me to make lifestyle changes’, which may be more likely to evoke a positive response when someone is asked whether or not they agree.

Patients were conscious of physical limitations or psychological morbidities demotivating efforts for lifestyle change, including musculoskeletal complaints, shortness of breath, chest pain, fear of recurrent cardiovascular events, and anxiety and depression. Participants discussed how patients could have different levels of motivation and beliefs or reasons for changing their physical activity or diet. It was suggested that these two lifestyle behaviours should be separated during discussions, as patients might respond differently to questions regarding each behaviour. However, this view was not universal and others described how both behaviours were inter-related and therefore important to consider together to motivate changes.

### Theme 4. Implementation in primary care

Views were expressed as to how the aid, particularly in a reduced format, could be implemented. The most common issue identified was a need for the aid to be available in a digital format. Participants discussed how, for its effective use in practice, SHARE-D would need to be integrated into electronic health record (EHR) systems. A number of GPs discussed how they would use tools such as QRISK3 with patients and ‘screen share’ to prompt discussions. The SHARE-D aid could be used in a similar manner. Some GPs and health professionals reported that the aid could be suitable for initiating discussions in response to abnormal test results, including elevated HbA1c or blood pressure. GPs also suggested it could be valuable during conversations with patients who are overweight. This was reflected in interviews that highlighted that, while weight was a topic that was often talked about during primary care consultations, SHARE-D could help in identifying support needed to make changes. Referring to some barriers to use that were identified (such as patients’ current readiness to make lifestyle change), it was highlighted how the flexible and adaptable nature of the aid meant even brief information could be useful to patients at a later date. Participants also suggested how patients could initially go through the aid themselves, prior to a consultation in which it would be discussed with a health professional. The potential for using the aid during telephone or virtual consultations was also discussed. This was influenced by the interviews taking place early during the COVID-19 pandemic, when there was increased use of telephone consultations. Participants also discussed how the aid could be used by other primary care staff, including practice nurses or physiotherapists, as well as by GPs.

### Changes made based on survey ratings and findings from qualitative interviews

Items with low agreement on inclusion (*n* = 11/39) were discussed at a consensus meeting between study authors. Two of these 11 items were retained following suggested modifications and nine were removed (see Table 4). Based on participants’ suggestions for clarification the wording of a further 11 items was amended, meaning that 17/39 (44%) items from the original aid had no, or only minor, changes to simplify wording. Key changes thus included a substantial reduction in the overall length of the aid by removing or reframing questions.

## Discussion

### Summary

This study used a mixed-methods approach to explore patient and health professional perspectives on a SDM aid (SHARE-D) for structuring brief lifestyle change conversations and to gain consensus on how it could be refined to improve implementation in primary care. Survey data identified components with low consensus for inclusion, including pictorial images and questions that were perceived as being similar to others and had no added value, mostly relating to the effects of different lifestyle choices. Individual interviews, focusing on these areas and on how SHARE-D could be most effectively delivered, pointed to it being suitable for initiating conversations following abnormal clinical findings and for promoting patient autonomy around healthy lifestyle choices. The support options provided, including a decision to discuss lifestyle change with others after a consultation, were seen as important. The aid was also regarded as useful for identifying potential barriers to engaging with lifestyle change, including fears around recurrent cardiovascular events, as well as musculoskeletal complaints associated with increased or unfamiliar levels of physical activity. Key barriers to use included structural and process issues such as the limited time during consultations and the paper-based format. Given workloads and time constraints in primary care, different approaches were suggested to improve implementation. This included developing a digital version that could be integrated into patient record systems. In addition, use by other staff, including practice nurses or physiotherapists working in primary care practices, as well as GPs, and patients initially going through the aid themselves prior to an appointment, were emphasised as methods to support delivery.

### Strengths and limitations

A strength of this study was that it examined a range of perspectives, including those of GPs, other health professionals, and patients. Although participants were recruited primarily from one geographical area of the UK, recruitment was purposeful and individuals from a range of settings and characteristics participated, meaning that findings could be transferable in a wider context. One potential limitation is that a formal consensus method, such as the nominal group process or Delphi technique, was not used. However, the mixed-methods approach, including individual interviews, reflecting on both individual and collated survey data, may have allowed for greater exploration of patient views, and for all perspectives to be examined in more depth than would be feasible with other group-based methods. Some interviews were conducted 2–3 weeks after completion of the survey, so this may have influenced recall of the aid. A further limitation is that the authors did not assess health literacy levels and this factor may have impacted on the perspectives of participants in this study. Higher health literacy has been associated with better experiences during SDM and improved capability wellbeing,^33^ which is the ability to do and be the things that matter to the individual.^34^

### Comparison with existing literature

These findings accord with previous research that developed and tested the feasibility of the SHARE-D aid.^23^ This study led to a consolidation and refinement of the core content of the aid and its presentation and use in primary care. The modifications that have been made to the aid as a consequence of the present study findings include reducing its length, simplifying content, and identifying acceptable approaches to its implementation. A reduction in the content of the SDM aid was made based on the level of agreement on item inclusion. An important consideration is that this could potentially moderate the efficacy of the aid. However, care was taken to ensure that modifications were reflective of patient and healthcare professional expert panel member perspectives and retained core components of the aid, particularly those related to international SDM tool development standards.^35^ This is particularly important for SDM aids used in primary care, since reducing content might have benefits in terms of improving the implementation, usability, and acceptability of a tool, relative to a more complex, in-depth tool.^36^ There may also be important differences SDM tools used for treatment choice decisions made in secondary care settings and those based around supporting chronic disease self-management, including lifestyle change approaches.

SHARE-D provides a meaningful structure for a focused discussion about lifestyle change in a time-efficient way that could support implementation in general practice. As a part of the ’making every contact count’ approach, NHS and general practice policy recommendations encourage the discussion of lifestyle change during routine patient interactions.^37–39^ One study has indicated that discussion aids can be successful in modifying conversations in primary care, leading to an increased number of discussions around physical activity, diet, and medications.^40^ In the present study, health professionals were keen to have lifestyle change discussions with patients and saw the value of doing so. Most felt equipped to initiate conversations, and felt they were sufficiently knowledgeable about current dietary advice guidelines and recommended levels of physical activity for patients with increased cardiovascular risk factors. Similar findings were reported in studies where primary care physicians reported having adequate knowledge and understanding about the health benefits for patients associated with achieving recommended levels of physical activity,^41^ even though other work has demonstrated that physical activity is less likely to be discussed with patients compared to other modifiable factors, such as smoking or blood pressure.^42^ While there is evidence to suggest that brief interventions can be effective in producing small but significant behavioural changes in patients,^43^ behaviour change conversations around physical activity can be challenging.^1,44^ Thus, standardised training may be necessary to provide clinicians with effective, evidence-based skills.^45,46^ Health professionals in the present study emphasised factors that limited the opportunity to have conversations about lifestyle, acknowledging that they often felt they lacked information on support options or resources to which patients might be referred, including details of local exercise groups or other relevant community-based organisations. Specific training in SDM and its use in managing chronic conditions have been suggested as potential components for decision aid standards.^35^

These findings also highlight the importance of involving patients in decisions about their own lifestyle to promote autonomy. Primary care patients’ knowledge, self-efficacy, perceived support from others. and perception that their choices and decisions were being taken into account have been identified as core factors influencing the adoption of healthy lifestyle change.^47–50^ This finding is in line with current draft National Institute for Health and Care Excellence guidelines that underline the importance of discussing risks, benefits, and consequences of care choices so that patients can make informed decisions.^51^ This guidance emphasises how decision making can take place before, during, and after appointments, and recommendations have been made to improve awareness, patient activation, and to embed SDM at an organisational level.^51,52^ This process may be facilitated by use of non-complex tools that integrate with existing clinic workflows, and which prompt users to engage so that they fully understand the role of SDM and are at ease with having brief but open discussions about lifestyle change.^53^ In this context, it is also important to ensure that the use of decision aids is equitable. There is evidence that regardless of multimorbidity, primary care patients in more deprived geographical areas have shorter consultation times;^54,55^ however, it has been demonstrated that allowing more time can be associated with increased patient autonomy and ability to self-manage conditions.^56^ It is therefore recommended that longer appointments, particularly for patients in areas of higher deprivation, are facilitated,^57^ and the brief and flexible nature of the SHARE-D aid supports this approach.

### Implications for research and practice

This mixed-methods study examined views of GPs, other health professionals, patients, and patient support workers on the SHARE-D aid and explored how it should be refined to improve implementation in primary care settings. The aid was seen as valuable and suitable for initiating brief conversations and promoting patient autonomy in primary care settings. Development of a digital version of the aid and use by other practice staff could minimise the impact on GP workloads. A previous study on digital SDM aids in cardiovascular disease management used standalone software.^17^ How to most effectively disseminate a digital version of the SHARE-D aid and integrate it within existing primary care systems requires further examination. One potential option would be to link the aid to EHR systems. The impact of SDM aids delivered via EHRs has previously been shown to be feasible and acceptable to users for the management of long-term conditions in a primary care context.^58^ Use of EHR-linked patient portals has also been associated with observed improvements in health status monitoring, patient–doctor interactions, overall care quality, and patient satisfaction around decision making;^59–61^ however, utilisation with these digital approaches in these studies may have been more closely associated with increased patient age profiles and with female sex.

Staff from different disciplines working in primary care can deliver brief lifestyle change advice to patients with increased cardiovascular risk factors. Better understanding of how such approaches and different models of care can be implemented is essential to remove organisational silos.^62,63^ Given resource and time constraints, studies are also needed to examine the effectiveness of using the SHARE-D aid in general practice, and to explore how SDM about lifestyle change can be best implemented. These studies need to assess outcomes for measuring effectiveness of implementation, including patient satisfaction and knowledge, decisional conflict and readiness, as well as associated costs, resource utilisation, and the impact on consultation times. Additional studies are also needed to assess how patient outcomes are influenced by the processes used to develop SDM aids for use in primary care contexts.

## References

[1] van der Wardt V, di Lorito C, Viniol A (2021). Promoting physical activity in primary care: a systematic review and meta-analysis. Br J Gen Pract.

[2] National Institute for Health and Care Excellence (2016). Cardiovascular disease: risk assessment and reduction, including lipid modification. CG181. https://www.nice.org.uk/guidance/cg181.

[3] O’Connor EA, Evans CV, Rushkin MC (2020). Behavioral counseling to promote a healthy diet and physical activity for cardiovascular disease prevention in adults with cardiovascular risk factors: updated evidence report and systematic review for the us preventive services task force. JAMA.

[4] Höchsmann C, Dorling JL, Martin CK (2021). Effects of a 2-year primary care lifestyle intervention on cardiometabolic risk factors: a cluster-randomized trial. Circulation.

[5] Schwalm J-. D, McCready T, Lear SA (2020). Exploring new models for cardiovascular risk reduction: the Heart Outcomes Prevention and Evaluation 4 (HOPE 4) canada pilot study. CJC Open.

[6] Bonner C, Raffoul N, Battaglia T (2020). Experiences of a national web-based heart age calculator for cardiovascular disease prevention: user characteristics, heart age results, and behavior change survey. J Med Internet Res.

[7] Chatterjee R, Chapman T, Brannan MG, Varney J (2017). GPs' knowledge, use, and confidence in national physical activity and health guidelines and tools: a questionnaire-based survey of general practice in England. Br J Gen Pract.

[8] Bardach SH, Schoenberg NE (2018). The role of primary care providers in encouraging older patients to change their lifestyle behaviors. Clin Gerontol.

[9] Hamilton K, Henderson J, Burton E, Hagger MS (2019). Discussing lifestyle behaviors: perspectives and experiences of general practitioners. Health Psychol Behav Med.

[10] Berman AH, Kolaas K, Petersén E (2018). Clinician experiences of healthy lifestyle promotion and perceptions of digital interventions as complementary tools for lifestyle behavior change in primary care. BMC Fam Pract.

[11] Ismail K, Bayley A, Twist K (2020). Reducing weight and increasing physical activity in people at high risk of cardiovascular disease: a randomised controlled trial comparing the effectiveness of enhanced motivational interviewing intervention with usual care. Heart.

[12] Murphy AW, Cupples ME, Smith SM (2009). Effect of tailored practice and patient care plans on secondary prevention of heart disease in general practice: cluster randomised controlled trial. BMJ.

[13] Albury C, Hall A, Syed A (2019). Communication practices for delivering health behaviour change conversations in primary care: a systematic review and thematic synthesis. BMC Fam Pract.

[14] Brinks J, Fowler A, Franklin BA, Dulai J (2016). Lifestyle modification in secondary prevention: beyond pharmacotherapy. Am J Lifestyle Med.

[15] Joseph-Williams N, Newcombe R, Politi M (2014). Toward minimum standards for certifying patient decision aids: a modified delphi consensus process. Med Decis Making.

[16] Wells S, Furness S, Rafter N (2008). Integrated electronic decision support increases cardiovascular disease risk assessment four fold in routine primary care practice. Eur J Cardiovasc Prev Rehabil.

[17] Peiris DP, Joshi R, Webster RJ (2009). An electronic clinical decision support tool to assist primary care providers in cardiovascular disease risk management: development and mixed methods evaluation. J Med Internet Res.

[18] Sutton RT, Pincock D, Baumgart DC (2020). An overview of clinical decision support systems: benefits, risks, and strategies for success. Nature.

[19] Bonner C, Fajardo MA, Doust J (2019). Implementing cardiovascular disease prevention guidelines to translate evidence-based medicine and shared decision making into general practice: theory-based intervention development, qualitative piloting and quantitative feasibility. Implement Sci.

[20] Menear M, Garvelink MM, Adekpedjou R (2019). Factors associated with shared decision making among primary care physicians: findings from a multicentre cross-sectional study. Health Expect.

[21] Vaisson G, Provencher T, Dugas M (2021). User involvement in the design and development of patient decision aids and other personal health tools: a systematic review. Med Decis Making.

[22] Witteman HO, Maki KG, Vaisson G (2021). Systematic development of patient decision aids: an update from the ipdas collaboration. Med Decis Making.

[23] Reed JR, Estabrooks P, Pozehl B (2019). Effectiveness of the 5a’s model for changing physical activity behaviors in rural adults recruited from primary care clinics. J Phys Act Health.

[24] Martínez C, Castellano Y, Andrés A (2017). Factors associated with implementation of the 5a’s smoking cessation model. Tob Induced Dis.

[25] Michie S, van Stralen MM, West R (2011). The behaviour change wheel: a new method for characterising and designing behaviour change interventions. Implement Sci.

[26] Barber CEH, Spencer N, Bansback N (2021). Development of an implementation strategy for patient decision aids in rheumatoid arthritis through application of the behavior change wheel. ACR Open Rheumatol.

[27] Cupples ME, Cole JA, Hart ND (2018). Shared decision-making (SHARE-D) for healthy behaviour change: a feasibility study in general practice. BJGP Open.

[28] Saldaña J, Leavy P (2020). The Oxford handbook of qualitative research.

[29] Tong A, Sainsbury P, Craig J (2007). Consolidated criteria for reporting qualitative research (COREQ): a 32-item checklist for interviews and focus groups. Int J Qual Health Care.

[30] Vasileiou K, Barnett J, Thorpe S, Young T (2018). Characterising and justifying sample size sufficiency in interview-based studies: systematic analysis of qualitative health research over a 15-year period. BMC Med Res Methodol.

[31] Nair R, Aggarwal R, Khanna D (2011). Methods of formal consensus in classification/diagnostic criteria and guideline development. Semin Arthritis Rheum.

[32] Warrens MJ (2010). Inequalities between multi-rater kappas. Adv Data Anal Classif.

[33] Xu RH, Zhou L-M, Wong EL-Y, Wang D (2021). The association between patients' eHealth literacy and satisfaction with shared decision-making and well-being: multicenter cross-sectional study. J Med Internet Res.

[34] Al-Janabi H, Flynn TN, Coast J (2012). Development of a self-report measure of capability wellbeing for adults: the icecap-a. Qual Life Res.

[35] Stacey D, Volk RJ (2021). The international patient decision aid standards (ipdas) collaboration: evidence update 2.0. Med Decis Making.

[36] Wallace BC, Jones J, Masoudi FA (2021). Development and piloting of four decision aids for implantable cardioverter-defibrillators in different media formats. Pacing Clin Electrophysiol.

[37] Lawrence W, Black C, Tinati T (2016). “Making every contact count”: evaluation of the impact of an intervention to train health and social care practitioners in skills to support health behaviour change. J Health Psychol.

[38] Chisholm A, Ang-Chen P, Peters S (2019). Public health practitioners’ views of the “making every contact count” initiative and standards for its evaluation. J Public Health.

[39] Health Education England (2018). Making every contact count: case studies. http://www.makingeverycontactcount.co.uk/implementing/case-studies.

[40] Boehmer KR, Dobler CC, Thota A (2019). Changing conversations in primary care for patients living with chronic conditions: pilot and feasibility study of the ican discussion aid. BMJ Open.

[41] Znyk M, Polańska K, Wojtysiak P (2019). Predictors of counselling related to a healthy lifestyle carried out by a general practitioner. Int J Environ Res Public Health.

[42] Wheeler PC, Mitchell R, Ghaly M, Buxton K (2017). Primary care knowledge and beliefs about physical activity and health: a survey of primary healthcare team members. BJGP Open.

[43] Patnode CD, Evans CV, Senger CA (2017). Behavioral counseling to promote a healthful diet and physical activity for cardiovascular disease prevention in adults without known cardiovascular disease risk factors: updated evidence report and systematic review for the us preventive services task force. JAMA.

[44] Speer SA, McPhillips R (2018). Initiating discussions about weight in a non-weight-specific setting: what can we learn about the interactional consequences of different communication practices from an examination of clinical consultations?. Br J Health Psychol.

[45] Wattanapisit A, Tuangratananon T, Thanamee S (2018). Physical activity counseling in primary care and family medicine residency training: a systematic review. BMC Med Educ.

[46] Lawrence W, Watson D, Barker H (2021). Meeting the UK government’s prevention agenda: primary care practitioners can be trained in skills to prevent disease and support self-management. Perspect Public Health.

[47] Jerdén L, Dalton J, Johansson H (2008). Lifestyle counseling in primary care in the united states and sweden: a comparison of patients’ expectations and experiences. Glob Health Action.

[48] Bourhill J, Lee JJ, Frie K (2021). What makes opportunistic gp interventions effective? An analysis of behavior change techniques used in 237 gp-delivered brief interventions for weight loss. Ann Behav Med.

[49] Yu CH, McCann M, Sale J (2021). “In my age, we didn’t have the computers”: using a complexity lens to understand uptake of diabetes ehealth innovations into primary care — a qualitative study. PLoS One.

[50] Coronado-Vázquez V, Canet-Fajas C, Delgado-Marroquín MT (2020). Interventions to facilitate shared decision-making using decision aids with patients in primary health care: a systematic review. Medicine.

[51] National Institute of Health and Care Excellence (2021). Shared decision making. NG197. https://www.nice.org.uk/guidance/indevelopment/gid-ng10120.

[52] Joseph-Williams N, Lloyd A, Edwards A (2017). Implementing shared decision making in the NHS: lessons from the magic programme. BMJ.

[53] Joseph-Williams N, Abhyankar P, Boland L (2021). What works in implementing patient decision aids in routine clinical settings? A rapid realist review and update from the International Patient Decision Aid Standards Collaboration. Med Decis Making.

[54] Stevens S, Bankhead C, Mukhtar T (2017). Patient-level and practice-level factors associated with consultation duration: a cross-sectional analysis of over one million consultations in English primary care. BMJ Open.

[55] Gopfert A, Deeny SR, Fisher R, Stafford M (2021). Primary care consultation length by deprivation and multimorbidity in England: an observational study using electronic patient records. Br J Gen Pract.

[56] Mercer SW, Fitzpatrick B, Gourlay G (2007). More time for complex consultations in a high-deprivation practice is associated with increased patient enablement. Br J Gen Pract.

[57] Mercer SW, O'Brien R, Fitzpatrick B (2016). The development and optimisation of a primary care-based whole system complex intervention (CARE Plus) for patients with multimorbidity living in areas of high socioeconomic deprivation. Chronic Illn.

[58] Fiks AG, Mayne SL, Karavite DJ (2015). Parent-reported outcomes of a shared decision-making portal in asthma: a practice-based RCT. Pediatrics.

[59] Carini E, Villani L, Pezzullo AM (2021). The impact of digital patient portals on health outcomes, system efficiency, and patient attitudes: updated systematic literature review. J Med Internet Res.

[60] Kinney AP, Sankaranarayanan B (2021). Effects of patient portal use on patient satisfaction: survey and partial least squares analysis. J Med Internet Res.

[61] Graham TAD, Ali S, Avdagovska M, Ballermann M (2020). Effects of a web-based patient portal on patient satisfaction and missed appointment rates: survey study. J Med Internet Res.

[62] Berman AH, Kolaas K, Petersén E (2018). Clinician experiences of healthy lifestyle promotion and perceptions of digital interventions as complementary tools for lifestyle behavior change in primary care. BMC Fam Pract.

[63] Chisholm A, Byrne-Davis L, Peters S (2020). Online behaviour change technique training to support healthcare staff 'Make Every Contact Count'. BMC Health Serv Res.

